# Mr. Kidd, on Hydrophobia

**Published:** 1810-06

**Authors:** W. L. Kidd

**Affiliations:** Surgeon, his Majesty's ship Raleigh. Downs


### *To the Editors of the Medical and Physical Journal.*

GENTLEMEN,

ABSENCE from England prevented my seeing, till a few days ago, your Journal for last July, containing the valuable paper of Dr. Valentin, on that greatest of human miseries, Hydrophobia, in which it is said that Professor

Rossi has published in the Memoirs of the Academy of Turin, contrary to the axiom of "*Nemo dat quod non habet*," the cases of three persons who died of hydrophobia, after being bit by cats which were not mad, but greatly irritated. I myself was a witness a few years ago of a case of this kind, but believing it to be a solitary one, I hesitated in communicating it to the public.

The child of a clergyman had a favourite cat, which was one day taken from him by his playmates, and hunted about by them till it was much irritated; the animal at length took shelter in a stable under the manger, from whence its pursuers could not dislodge it; the boy who owned it, wishing to rescue his favourite, put his hand and arm into its lurking place to lay hold of it, but soon withdrew them on finding his hand severely bitten and scratched. I believe nothing more was done for the child than to dress the wounds till they healed. In a short time after, I think in about three weeks, he was seized with the usual symptoms of hydrophobia, and died.

Might not hydrophobia, whether human, canine, or feline, be considered as a disease of great irritation produced by a variety of causes, the principal of which is the absorption of the saliva of an animal labouring under the same disease? If this would be a proper definition, it would not be a contradiction of the above quoted axiom.

If any credit can be attached to the cures of this disease by vinegar, mentioned in the same paper, p. 61, and I should hope that on a subject such as this, nothing untrue would be willingly inserted, I think they afford great encouragement to British practitioners to give that remedy a trial; but surely, if such a cure as that said to have taken place eleven years ago at Chaumerac, had really occurred, not even so powerful a preventive as hostilities between the two countries, would have hindered such "glad tidings" from reaching us. The attention of the medical world, however, seems now so laudably turned towards the cure of this dire malady, that I have strong hopes of shortly seeing it scratched off the list of diseases that stand their ground at present, at once a scourge to the world, and a disgrace to the medical profession.

I am, &c.

W. L. KIDD,

Surgeon, his Majesty's ship Raleigh.

*Downs,*

*April* 20, 1810.

